# Highly tunable low frequency metamaterial cavity for vibration localization

**DOI:** 10.1038/s41598-022-13453-1

**Published:** 2022-06-11

**Authors:** Hong Woo Park, Hong Min Seung, Wonjae Choi, Miso Kim, Joo Hwan Oh

**Affiliations:** 1 Department of Mechanical Engineering, Ulsan National Institute of Science and Technology, UNIST-Gil 50, Eonyang-eup, Ulju-gun, Ulsan, 44919 Republic of Korea; 2 Department of Science of Measurement, University of Science and Technology (UST), Gajeong-ro 207, Yuseong-gu, Daejeon, 34113 Republic of Korea; 3 AI Metamaterial Research Team, Korea Research Institute of Standards and Science, Gajeong-ro 267, Yuseong-gu, Daejeon, 34113 Republic of Korea; 4 School of Advanced Materials Science and Engineering, Sungkyunkwan University, Seobu-ro 2066, Jangan-gu, Suwon, 16419 Republic of Korea

**Keywords:** Mechanical engineering, Acoustics

## Abstract

Metamaterial cavity has gathered much attention recently due to its capability of localizing vibration energy. Despite the active research, however, there are still big technical challenges not solved yet. Especially, there has been no approach to maximize the wave localization performance of metamaterial cavity; therefore, there has been a possibility that obtained cavity mode does not show sufficiently high performance. Also, there is a tunability issue that whole metamaterials should be re-designed to tune the cavity frequency. Here, we present the metamaterial cavity system that can control its cavity mode frequency from 589 to 2184 Hz by adjusting the cavity length from 140 to 60 mm without re-designing the whole metamaterial based on the broad bandgap. Also, the performance of the obtained cavity mode can be improved by adjusting the length of the side beam attached to the metamaterial; the displacements are amplified more than 18–110 times. Consequently, one may easily obtain the highly localized vibration energy at the desired frequency by adjusting two geometric parameters based on the proposed metamaterial cavity system. Numerical and experimental supports are provided to validate our new metamaterial cavity system. This metamaterial cavity system is expected to provide a guideline for localizing vibration energy in various applications, such as energy harvesting, sensing or vibration dissipation.

## Introduction

In the last few decades, metamaterials consisting of artificially designed unit cells have opened a new way to manipulate wave and vibration, which was impossible with the conventional approaches. Metamaterials have been vigorously studied in various applications, such as bandgap preventing wave propagation^1–4^, refracting wave in a negative refraction angle^5–9^, achieving diode-like behavior of wave^10–13^, or overcoming the diffraction limit and achieving super-resolution^14–17^. Among these fascinating phenomena, metamaterial cavity has been gathered much attention due to its capability of wave (or vibration) energy manipulation. By locally breaking periodicity of metamaterials with bandgap, wave (or vibration) energy can be highly localized inside the cavity. Thus, metamaterial cavity has been widely applied in waveguiding^18–20^, or amplifying the vibration^21,22^. In particular, improving the performance of energy harvesting by localizing vibration energy with metamaterial cavity has attracted more attention nowadays^23–30^.

Here, let us review the previous representative studies on the metamaterial cavity for localizing vibrational energy. Lv et al. showed that elastic vibration energy harvesting could be enhanced by localizing the elastic vibration within the cavity through the phononic crystal consisting of periodically arranged steel rods within rubber^23^. Jo et al. provided an analytic model for phononic crystal with a piezoelectric defect for energy harvesting^24^, and showed the effect of the supercell size and defect location on the efficiency of energy harvesting by numerical simulations^25^. Park et al. experimentally showed that the cavity on a thin aluminum phononic crystal could improve the energy harvesting efficiency about 22.8 times than the bare plate^26^. Wen et al. introduced topological cavities for robust energy harvesting and demonstrated its harvesting efficiency about 30 times than the bare plate and robustness against defects at moderate perturbation level^27^. However, in these studies, the cavity mode has a limitation that the amplification through the cavity mode operates in a single frequency due to its resonance nature. As an effort to overcome this limitation, Jo et al. introduced double defects to split the cavity mode and widen operating frequency^28,29^. Ma et al. exploited the high order modes of the cavity on an aluminum phononic crystal to obtain multiple cavity modes at various frequencies with a single metamaterial^30^. Lv et al. achieved two cavity modes at two separated bandgaps with a single phononic crystal consisting of aluminum and acrylic^31^.

Although a number of studies have been conducted, there are still various issues in the metamaterial cavity. Among them, the most critical and scientifically important issue is that the metamaterial cavity does not always guarantee better performance. In fact, previous research on metamaterial cavity has mainly focused on how to achieve the cavity, not on how to optimize its performance. Thus, there is a possibility that the vibration localization in a metamaterial cavity is not significantly better (or even worse) than the normal beam case. Obviously, if it happens, it is no use to design and achieve a metamaterial cavity. Unfortunately, a study focusing on the performance of metamaterial cavity have been extremely rare. Furthermore, previously proposed metamaterial cavities have been usually suffered from the narrow bandgap issue. In general, metamaterial’s bandgap is not broad enough to tune the cavity frequency in a broad range. Thus, the whole system including metamaterials should be re-designed to tune the cavity frequency, which is almost impossible in real applications. Nevertheless, there has been no attempt to achieve a metamaterial cavity having such high tunability of its frequency and performance.

In this paper, a metamaterial cavity system having high tunability of its frequency and performance is proposed by adapting a metamaterial having a broad bandgap and attaching an additional external resonator at the metamaterial (in this paper, the side beam). Recently, Park et al. provided a broad flexural bandgap, whose normalized bandwidth is 1.505, in a low-frequency regime by introducing a cylindrical mass and bow-tie shaped spring^32^. By adopting this design concept, we design a metamaterial with a bandgap from 522 to 3117 Hz (normalized bandwidth is 1.426). Then the cavity modes of various frequencies are obtained from 592 to 2317 Hz by changing the cavity length without the re-designing metamaterial. In addition, to optimize the metamaterial cavity’s performance, we focused on the evanescent wave coupling—we found that although the wave is localized inside the metamaterial cavity due to the bandgap, there exists coupling between the cavity and external structure due to the evanescent leaky waves. Thus, by introducing additional structure outside the metamaterial cavity (which we call side beam here), the performance of the metamaterial cavity can be optimized. Especially, in our metamaterial cavity, the cavity mode is formed at an extremely low frequency regime where wave decaying is weak so that evanescent wave coupling is quite active. As a result, the wave localization can be further enhanced by tuning the side beam’s resonance. The proposed metamaterial allows one can easily obtain the highly localized cavity mode at the desired frequency without concerning about the re-designing and amplification issues.

This paper is organized as follows. First, we review the condition of the unit cell for broad and low-frequency bandgap through an extended mass-spring system and propose the unit cell design. Second, the metamaterial cavity at various frequencies is designed with the same metamaterial to show that our metamaterial cavity has high tunability. Then the optimization method is shown by adjusting the side beam. Finally, realization and experimental validation of the designed metamaterial cavities are shown.

## Metamaterial with low frequency broad bandgap

Since bandgap is necessary to achieve the cavity mode, a broad bandgap at the low-frequency range is required to form a cavity at various low frequencies. Thus, before explaining details of the proposed metamaterial cavity, metamaterial with low-frequency broad bandgap is designed first. To clarify the design process, it is worth reviewing our theoretic model^33^ for analyzing flexural wave propagation to determine the condition of the broad and low-frequency bandgap.

**Figure 1:**
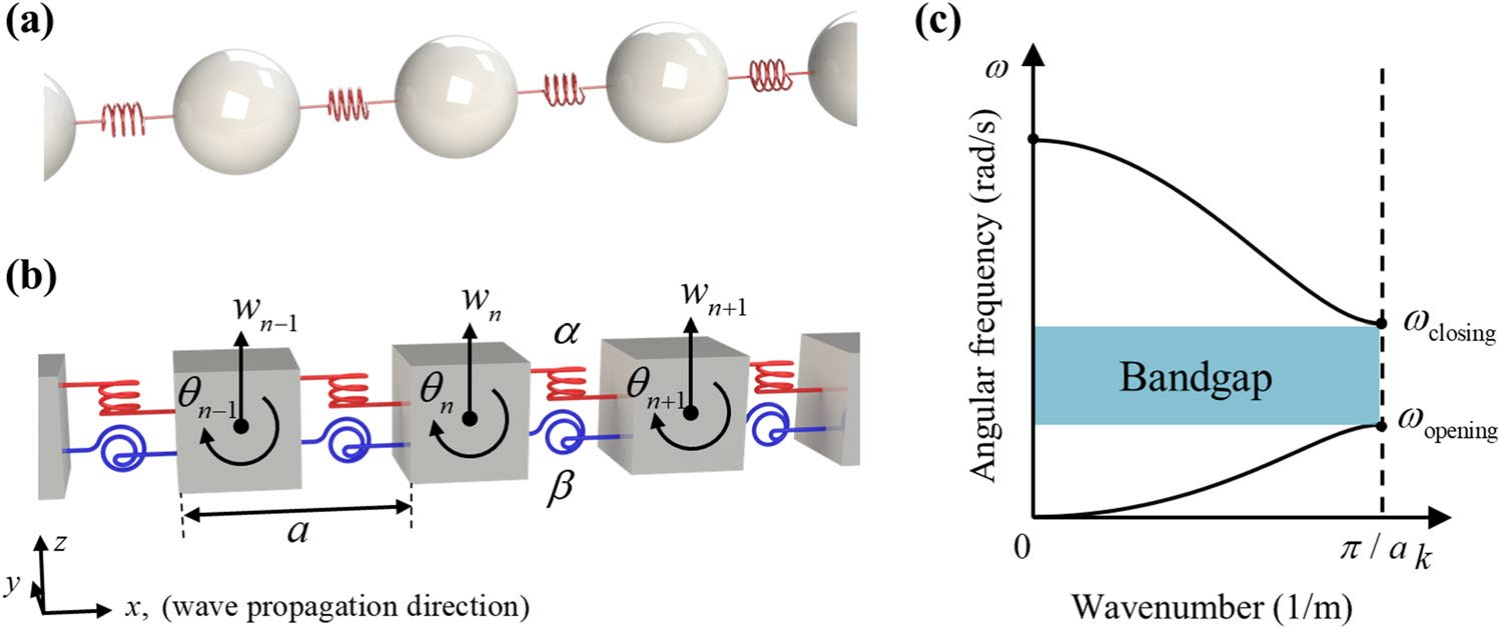
(**a**) A general mass-spring system. (**b**) The extended mass-spring system for analyzing flexural wave propagation. (**c**) Analytically calculated dispersion curve of the extended mass-spring system.

In general, the periodic mass-spring system, shown in Fig. 1a, has been commonly used to analyze wave propagation in metamaterials. Unfortunately, flexural wave is hard to be explained with the general mass-spring system. Instead, the extended mass-spring system, shown in Fig. 1b, has been used to consider the flexural wave propagation in metamaterial^32–35^. According to the classical Timoshenko beam theory, the flexural wave can be described by not only vertical displacement but also rotational motion^36^. To describe these two degrees of freedom, the extended mass-spring system consists of two kinds of springs and inertia—the spring for shear stiffness $\alpha$, the spring for bending stiffness $\beta$, mass *m* and rotational inertia *I* are periodically arranged with the periodicity *a*. In Fig. 1b, the z-directional displacement of the *n*th unit cell is denoted as *w*_*n*_, and the rotation angle of the *n*th unit cell is denoted as $\theta_{n}$. The governing equation of the *n*th mass in the extended mass-spring system can be written as

1a $$ m\frac{{\partial^{2} w_{n} }}{{\partial t^{2} }} = \alpha \left( {w_{n + 1} + w_{n - 1} - 2w_{n} } \right) + 0.5a\alpha \left( {\theta_{n + 1} - \theta_{n - 1} } \right), $$

1b $$ I\frac{{\partial^{2} \theta_{n} }}{{\partial t^{2} }} = \beta \left( {\theta_{n + 1} + \theta_{n - 1} - 2\theta_{n} } \right) - 0.5a\alpha \left( {w_{n + 1} - w_{n - 1} } \right) - (0.5a)^{2} \alpha \left( {\theta_{n + 1} + \theta_{n - 1} + 2\theta_{n} } \right). $$

Since this system is a periodic system, the Floquet–Bloch condition can be applied on the displacement and rotational angle; thus, the $n - 1$th term and $n + 1$th term can be replaced by the *n*th term as

2 $$ \begin{gathered} w_{n + 1} = e^{ - ika} w_{n} {,}\;\; \, w_{n - 1} = e^{ika} w_{n} , \, \hfill \\ \theta_{n + 1} = e^{ - ika} \theta_{n} {, }\;\;\theta_{n - 1} = e^{ika} \theta_{n} . \hfill \\ \end{gathered} $$

Substituting Eq. (2) into Eq. (1) yields

3a $$ - m\omega^{2} w_{n} = \alpha \left( {e^{ - ika} + e^{ika} - 2} \right)w_{n} + 0.5a\alpha \left( {e^{ - ika} - e^{ika} } \right)\theta_{n} , $$

3b $$ - I\omega^{2} \theta_{n} = \beta \left( {e^{ - ika} + e^{ika} - 2} \right)\theta_{n} - 0.5a\alpha \left( {e^{ - ika} - e^{ika} } \right)w_{n} - \left( {0.5a} \right)^{2} \alpha \left( {e^{ - ika} + e^{ika} + 2} \right)\theta_{n} . $$

The exponential terms in Eq. (3) can be expressed in trigonometric functions by Euler’s equation as below

4 $$ e^{ - ika} + e^{ika} = 2\cos \left( {ka} \right) \, \quad {\text{and}} \, \quad e^{ - ika} - e^{ika} = - i2\sin \left( {ka} \right). $$

Using Eq. (4), Eq. (3) can be expressed as

5a $$ - m\omega^{2} w_{n} = 2\alpha \left( {\cos \left( {ka} \right) - 1} \right)w_{n} - i\alpha a\sin \left( {ka} \right)\theta_{n} , $$

5b $$ - I\omega^{2} \theta_{n} = 2\beta \left( {\cos \left( {ka} \right) - 1} \right)\theta_{n} + i\alpha a\sin \left( {ka} \right)w_{n} - 0.5a^{2} \alpha \left( {\cos \left( {ka} \right) + 1} \right)\theta_{n} . $$

Equation (5) can be described in the matrix form as

6 $$ \left[ {\begin{array}{*{20}l} {2\alpha \left( {\cos \left( {ka} \right) - 1} \right) + m\omega^{2} } & {\quad - i\alpha a\sin \left( {ka} \right)} \\ {i\alpha a\sin \left( {ka} \right)} & {\quad 2\beta \left( {\cos \left( {ka} \right) - 1} \right) - 0.5a^{2} \alpha \left( {\cos \left( {ka} \right) + 1} \right) + I\omega^{2} } \\ \end{array} } \right]\left[ {\begin{array}{*{20}c} {w_{n} } \\ {\theta_{n} } \\ \end{array} } \right] = \left[ {\begin{array}{*{20}c} 0 \\ 0 \\ \end{array} } \right] $$

From the Eq. (6), the dispersion relation can be obtained by assuming the determinant of the matrix is zero to avoid a trivial solution, i.e., $w_{n} = \theta_{n} = 0$.

From the dispersion relation, we can obtain bandgap opening frequency and closing frequency, as can be seen in Fig. 1c. These frequencies can be easily calculated by substituting $k = \pi /a$ into Eq. (6) as

7 $$ \omega_{opening} = 2\sqrt {\beta /I} {, }\omega_{closing} = 2\sqrt {\alpha /m} . $$

From these results, it can be seen that the opening frequency $\omega_{opening}$ becomes lower by increasing rotational inertia *I* and decreasing bending stiffness $\beta$, while the closing frequency $\omega_{closing}$ becomes higher by increasing shear stiffness $\alpha$ and decreasing mass *m*. Thus, if one can design a spring structure with high shear stiffness and low bending stiffness, and design a mass structure with high rotational inertia and low mass, broad bandgap at low frequency is feasible.

**Figure 2:**
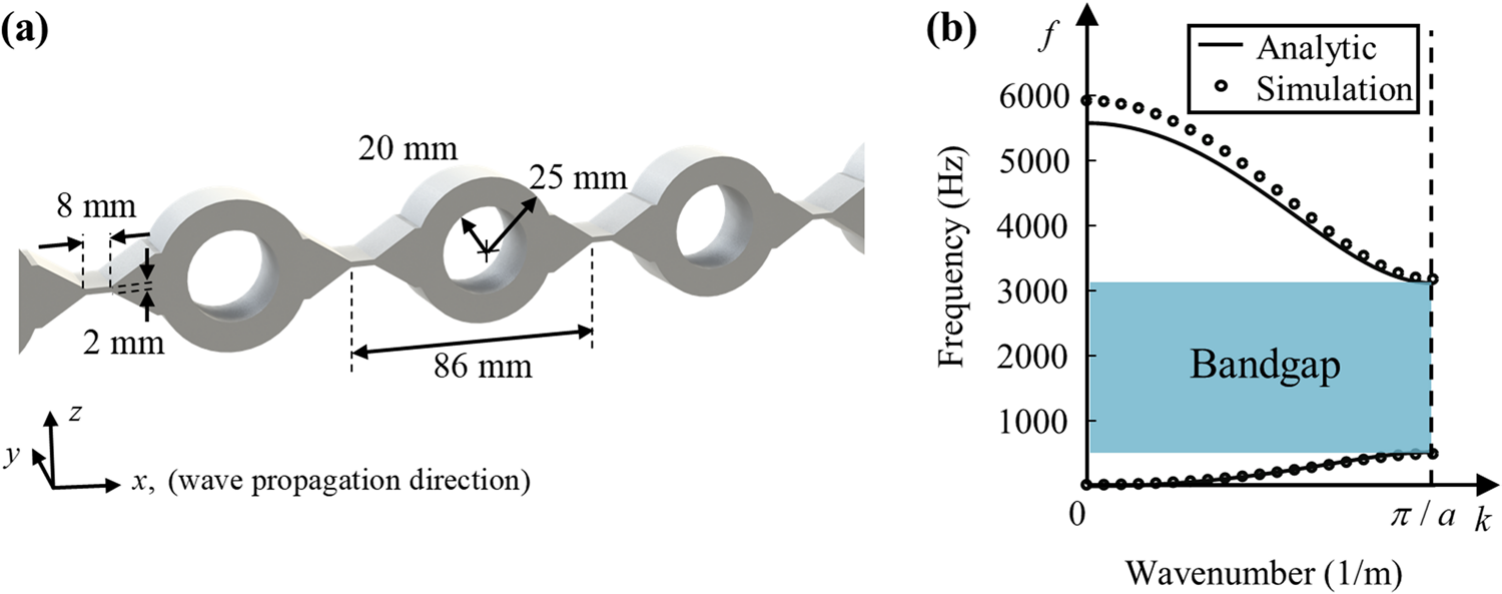
(**a**) Proposed metamaterial unit cell design for broad and low-frequency bandgap. (**b**) Analytic and numerical dispersion curves of the proposed unit cell design.

Previously, Park et al. introduced the hollow cylindrical mass and bow-tie shaped spring structure to achieve these goals^32^. However, the bow-tie shaped spring structure in the previous research had a curved shape, which is not preferred in the metamaterial cavity where a distinct distinguishment between metamaterial and cavity region is essential. Thus, in this work, a unit cell for the broad and low-frequency bandgap is newly designed with the angled bow-tie shaped structure as shown in Fig. 2a. The unit cell is made of aluminum, where the density is 2700 kg/m^3^, Young’s modulus is 70 GPa, and Poisson’s ratio is 0.33. It is composed of the hollow cylindrical mass, whose outer radius is 25 mm and inner radius is 20 mm, and bow-tie shaped spring with the thinnest thickness of 2 mm. These mass and spring structures with 20 mm depth in the y-direction are periodically arranged with the periodicity of 86 mm. The mechanical properties of the designed unit cell, shear stiffness, bending stiffness, mass, and rotational inertia, are calculated as $m = 81.053{\text{e}} {-} 3\, {\text{kg}}$, $I = 46.8{\text{e}}{-}6\,{\text{ kg}} \cdot {\text{m}}^{{2}}$, $\alpha = {7.77}{\text{e}}6\, {\text{N/m}}$, $\beta = 126.6\,{\text{N}} \cdot {\text{m/rad}}$ (these values are calculated from numerical simulations).

From these mechanical properties, the dispersion curve can be analytically obtained by substituting the properties into Eq. (6) and taking the determinant to be zero. As a result, the analytic dispersion curve plotted as a solid line in Fig. 2b can be obtained. From the dispersion curve, it can be clearly seen that the broad and low-frequency bandgap is achieved from 522 to 3117 Hz (the normalized bandwidth is 1.426) as desired. This result can be validated by comparing the analytic dispersion with the numerical one, plotted as a circle in Fig. 2b. As the two dispersion curves agree well with each other, it can be said that the extended mass-spring system used to analyze the flexural wave propagating through the beam is valid. Also, since the metamaterial has a broad bandgap, it is possible to install the cavity at various low frequencies from 522 to 3117 Hz without re-designing the metamaterial. As mentioned earlier, this provides the desired tunability.

## Metamaterial cavity for vibration localization at broad low frequency range

**Figure 3:**
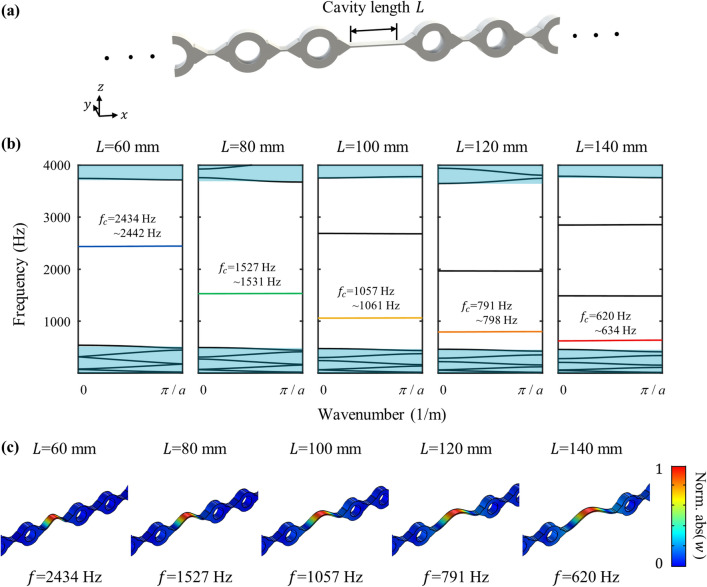
(**a**) Metamaterial cavity with the cavity length *L*. (**b**) Dispersion curves correspond to the cavity lengths of 60 mm, 80 mm, 100 mm, 120 mm and 140 mm. (**c**) Mode shape of the lowest order cavity mode for each cavity lengths.

Based on the designed metamaterial, the metamaterial cavity for vibration localization can be achieved at various low frequencies. It can be achieved by introducing a thin plate, denoted as the cavity, in Fig. 3a, inside the periodically arranged metamaterial. As the cavity is introduced, the periodicity is locally broken, so that a cavity mode is formed. This cavity mode can be checked by evaluating the wave dispersion curve of the supercell^23,29,30^. First, the supercell consisting of 5-unit cells (same as the one in Fig. 2a) with cavity inside is modeled with the Floquet condition at each side, same as calculating dispersion curves. The cavity has the length of arbitrary value *L* in x-direction, the thickness of 2 mm in z-direction and the depth of 20 mm in the y-direction. After that, the eigenfrequency analyses are carried out for various wavevectors to calculate the wave dispersion curve of the unit cell. Due to the cavity, a flat branch is newly formed inside the bandgap frequency range of the dispersion curve. Since the wave cannot exist inside the metamaterial due to the bandgap phenomenon, the wave can only exist inside the cavity; the vibration energy is localized inside the cavity. Therefore, one can figure out whether the metamaterial cavity is formed with the corresponding resonance frequency by calculating the dispersion curve of the supercell.

Since our goal is achieving the localization of vibration energy at various low frequencies with the same metamaterial, we used same metamaterial but different cavities targeted for different frequencies, respectively. To this end, we choose five cavity lengths, *L* = 60 mm, 80 mm, 100 mm, 120 mm and 140 mm. Then the dispersion curves of each cavity length are calculated, as in Fig. 3b. Note that only the flexural wave modes are plotted in Fig. 3b. It can be clearly seen that several cavity modes are successfully generated inside the bandgap, 522–3117 Hz. Among them, the lowest order flexural cavity modes, which is the main interest generally, are observed at the cavity frequencies *f*_*c*_ = 2434–2442 Hz, 1527–1531 Hz, 1057–1061 Hz, 791–798 Hz, and 620–634 Hz, corresponding to the cavity length 60 mm, 80 mm, 100 mm, 120 mm and 140 mm, respectively. Also, the lowest order cavity mode shape of each length can be checked by drawing z-directional displacement *w*, as shown in Fig. 3c. (In the rest of the paper, the z-directional displacement will be referred to as displacement for convenience.) It can be clearly seen that the lowest order flexural mode is formed with the cavity, validating that our metamaterial cavity provides vibration localization at various low frequencies. Note that since the cavity mode originates from the cavity’s resonant mode, as the cavity length is shortened, the cavity mode appears in the higher frequency.

## Effect of side beam on the cavity mode

In the previous section, metamaterial cavity is successfully formed by using the broad bandgap. However, how can one optimize the metamaterial cavity’s performance? In theory, waves cannot propagate through metamaterial at bandgap frequency since an infinite-sized metamaterial layer is assumed. However, in a real metamaterial cavity, the finite-sized metamaterial layer is introduced to form a cavity. Thus, a very small leaky wave is transferred through the metamaterial as evanescent waves. Especially in a low-frequency regime, the leaky wave is more actively formed because the decaying rate of evanescent modes is too low. As a result, the evanescent wave coupling through the metamaterial, the coupling between the cavity and the structure outside the metamaterial, exists. Consequently, we can manipulate the cavity’s performance by utilizing this evanescent wave coupling. In general, the coupling is weak so that it does not highly affect the cavity’s performance. However, if there exists resonance at the outside structure, the cavity’s performance is highly affected despite the weak coupling effect.

**Figure 4:**
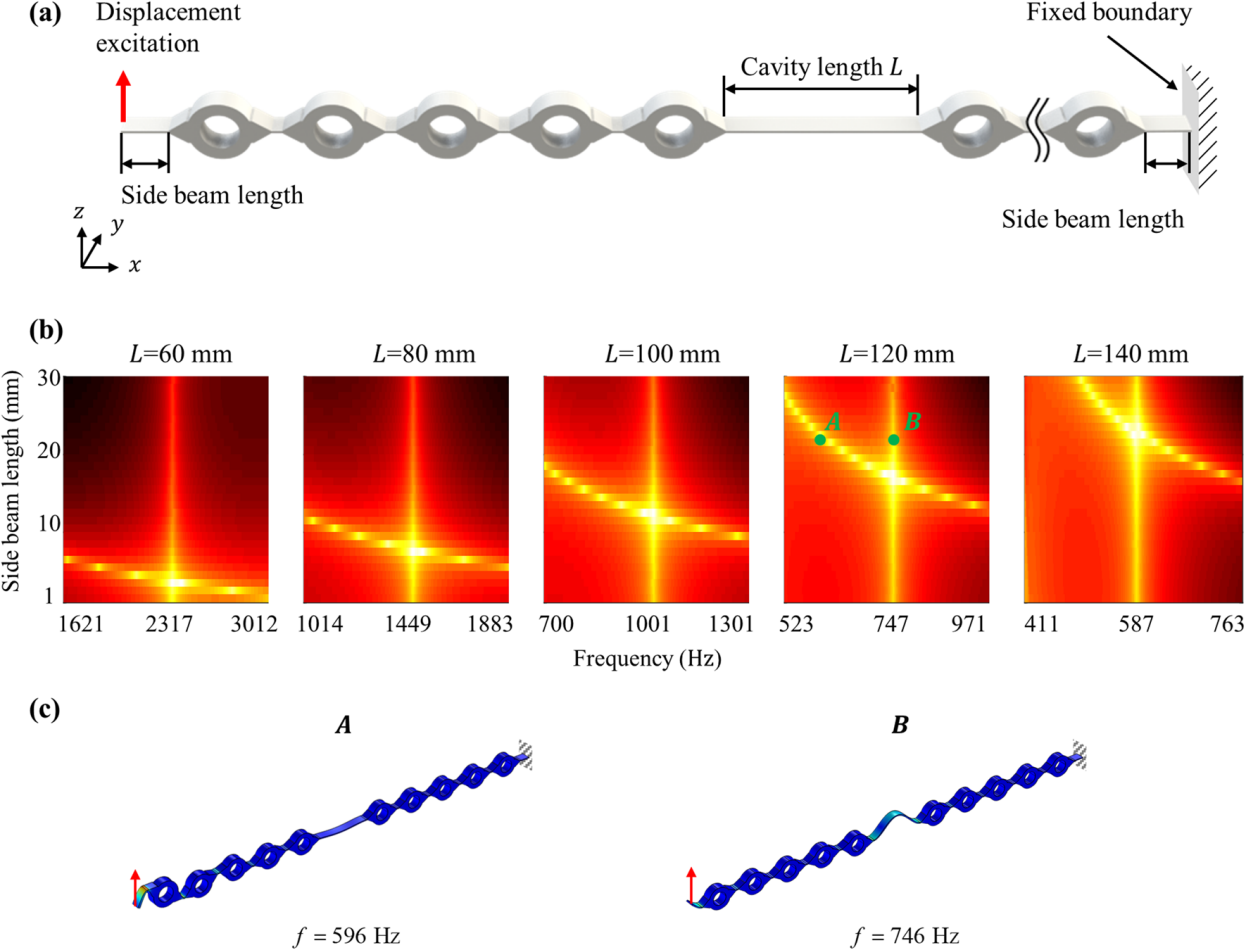
(**a**) Schematics of metamaterial system consisting of unit cell, cavity, and side beam. (**b**) Displacement of the cavity of 60 mm, 80 mm, 100 mm, 120 mm and 140 mm with varying frequency and side beam length. (**c**) Mode shape of metamaterial at the frequency of side beam resonances (A) and at the cavity mode (B).

Based on this, we added an additional beam, called a side beam, outside the metamaterial, as shown in Fig. 4a. Due to the finite length of the side beam, it has its own resonance frequency. Thus, the metamaterial cavity has two resonance frequencies—the resonance frequency of the cavity, and that of the side beam. Accordingly, we tuned the length of the side beam so that the resonance frequency of the side beam becomes almost the same as the resonance of the cavity. By doing so, when the flexural vibration of the cavity mode frequency incident to the metamaterial, the flexural vibration would get pumped up and more energy is transferred to the cavity by the evanescent wave coupling. This results in more vibration energy transfer into the cavity mode.

To support the idea, numerical simulations are carried out. In general, wave simulation using PML (perfectly matched layer) to prevent undesired reflected waves has been used for metamaterial cavities. However, since cavity modes are formed at low frequencies in our study, extremely large PML should be introduced so that conventional wave simulation is hard to be applied in the current metamaterial cavity. Thus, as shown in Fig. 4a, the vibration simulation without PML is considered instead. First, the side beam, which has the same height and depth as the cavity beam, is attached to both ends of the metamaterial. The side beam on the left is excited in the constant amplitude, while the other side is fixed. Note that the side beam has the same thickness in z-direction and depth in y-direction as the cavity beam. The normalized displacement at the center of the cavity is measured and plotted as shown in Fig. 4b with respect to the frequency and the side beam’s length. Note that a very small loss of the loss factor 0.01 is imposed to avoid any numerical issue occurred by the infinite displacement around the resonance frequency.

For each cavity length, the displacement at the center of the cavity is numerically calculated. In Fig. 4b, there are two highlighted lines, one is a vertically straight line, and the other is a horizontally curved line. The vertically straight line indicates the amplification due to the cavity mode. It can be seen that the vertically straight lines are located at the cavity’s resonance frequency, *f*_*c*_ = 2317 Hz, 1450 Hz*,* 1002 Hz, 747 Hz and 592 Hz for *L* = 60 mm, 80 mm, 100 mm, 120 mm, and 140 mm, respectively. On the other hand, the horizontally curved line shows the amplification due to the resonance of the side beam. It can be more clarified by mode shapes at the curved line (denoted as the green point A) and the straight line (denoted as the green point B) as shown in Fig. 4c. (The mode shapes are obtained from the metamaterial with *L* = 120 mm.) The mode shape at point A shows that the major deformation takes place at the side beam, indicating that the peak corresponds to the side beam’s resonance. As explained, although the resonance belongs to the side beam, high displacement is measured at the cavity due to the evanescent wave coupling between the cavity and the side beam.

Since the side beam’s resonance affects the cavity’s displacement, the cavity’s performance can be easily optimized by adjusting the length of the side beam. In Fig. 4b, it can be seen that the displacement becomes maximum value when the vertical straight line and the horizontally curved line intersect. In other words, if the resonance frequencies of the cavity and the side beam are almost the same, the metamaterial cavity’s performance is maximized. In the current metamaterial cavity, the optimal performance is achieved with the side beam length of 3 mm, 7 mm, 12 mm, 17 mm and 22 mm for *L* = 60 mm, 80 mm, 100 mm, 120 mm and 140 mm, respectively. One may wonder why the cavity and side beam have quite different lengths even though they have almost the same resonance frequencies. The reason is that a few unit cells nearby the incident point are involved in the resonance of the side beam, as shown in Fig. 4c, while there is almost no involvement of unit cells in the cavity’s resonance, as shown in Fig. 4d. The involvement of a few unit cells results in the effect of adding mass, and therefore the low resonance frequency is achieved despite the short length of side beams.

**Figure 5:**
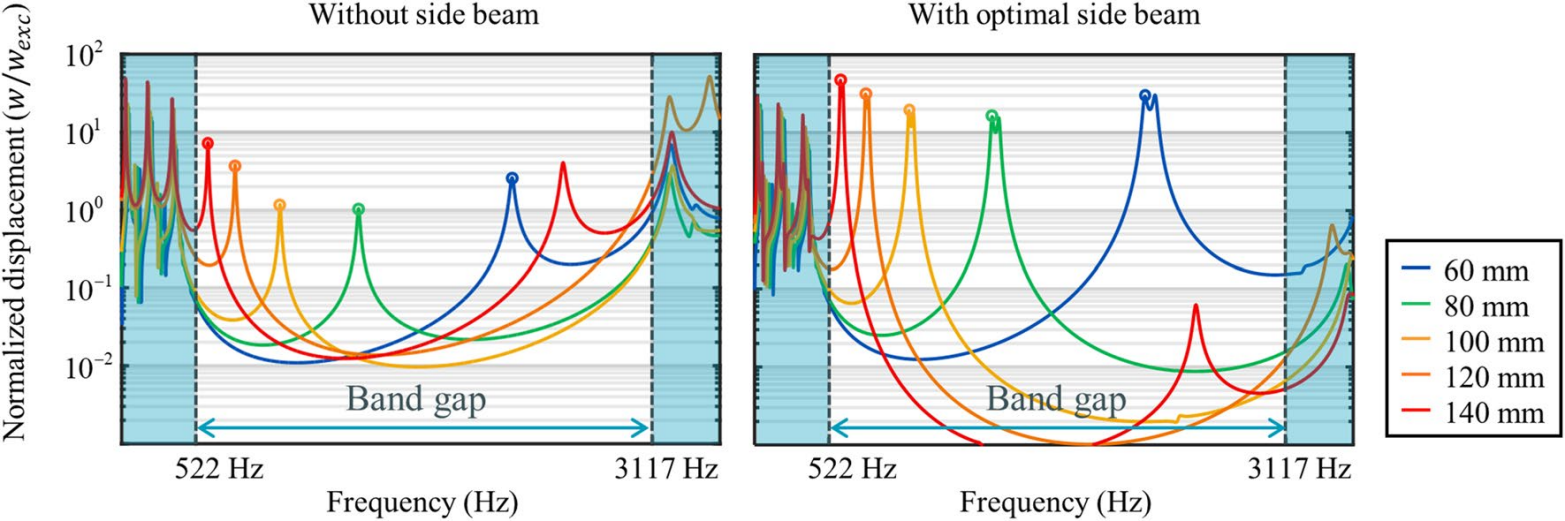
Normalized displacements for different cavity lengths without side beam (left) and with optimal side beam (right).

To further validate the effect of the side beam on the cavity mode, the normalized displacement of the metamaterial with and without the side beam is numerically calculated as shown in Fig. 5. To this end, two numerical models are considered; one is the metamaterial cavity in Fig. 4a with the optimal side beams, and the other one has no side beam. For both cases, the same simulations as in Fig. 4a are carried out; the left end of the system is excited with a harmonic displacement of constant amplitude while the right end of the system is fixed. The normalized displacement, which is obtained as the displacement at the middle of the cavity *w* divided by the displacement of the excitation point *w*_*exc*_, is calculated from 100 to 3500 Hz for each cavity length. As shown in Fig. 5, it can be clearly seen that the amplification ratio for every cavity length is significantly improved by attaching the side beam.

There are some worthwhile points to notice. First, the amplification of the cavity length of 80 mm and 100 mm is less than the amplification of the normal beam (the amplification of the normal beam of the same length is about 7–9. It will be addressed later.) As shown here, even though the cavity mode is formed, the amplification at the cavity mode might not be enough for practical applications. Although the high tunability in cavity mode frequency has been obtained, there is not useful if the performance improvement is not accompanied. However, the normalized displacements with the side beam exceed at least 10 for every cavity length. Second, two peaks exist inside the bandgap frequencies for the cavity length of 140 mm. The second peak, around 2610 Hz, is the higher order resonance mode of the cavity. In general, higher order mode is hard to be observed in the metamaterial cavity. However, since our metamaterial provides a very broad bandgap, the higher order resonance mode also can appear inside the bandgap frequency range. Lastly, it should be noted that the optimal length of the side beam changes as the boundary or force conditions changes. Here, the optimal length is defined under the boundary and force conditions shown in Fig. 4a. Therefore, if these conditions change, the optimal length also changes. Nevertheless, the results show that our metamaterial allows one can achieve the cavity mode, adjusting the cavity’s resonance frequency with the cavity length based on the broad bandgap and optimizing the cavity’s performance with the side beam length.

In addition, current research focused on the resonance frequencies of two resonances, the cavity and the side beams. However, since two resonators are involved, there is a possibility that the cavity’s performance can be further enhanced with the Q factor of each resonance. Since the metamaterial considered in the current research is not sufficient to study the Q factor effect, (since the Bragg gap usually exhibits low Q factors, compared to the resonance gap), this was not studied here. However. We’d like to comment that further research could be made on this point.

## Experiment and validation

**Figure 6:**
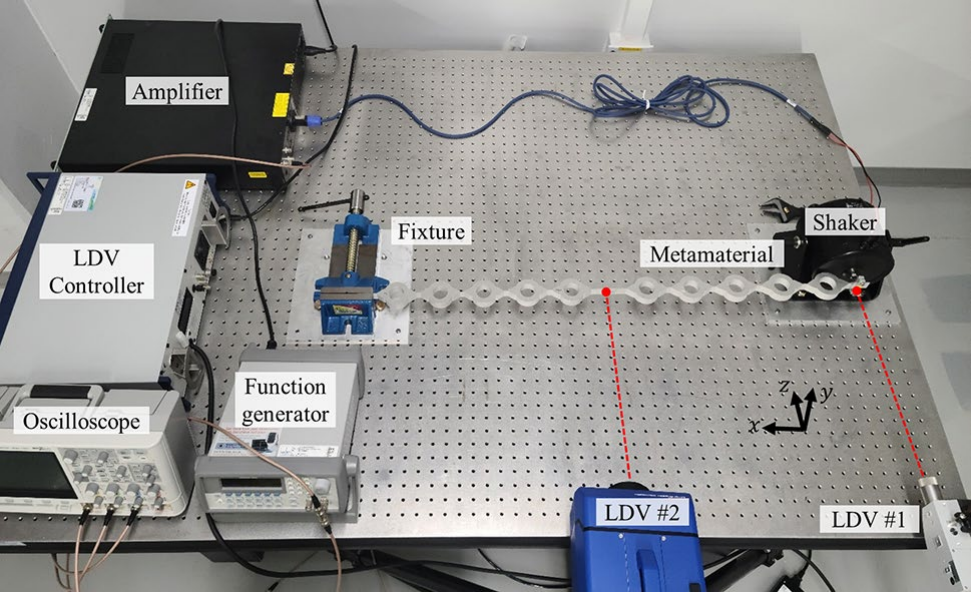
The overall experiment setup.

Finally, proposed metamaterial cavities are fabricated, and their performances are experimentally investigated. The fabricated metamaterial cavity and experimental settings are shown in the photo in Fig. 6. The metamaterials of cavity length 60 mm, 80 mm, 100 mm, 120 mm and 140 mm are fabricated by waterjet cutting with a bulk aluminum block. They have their optimized side beam, which is 3 mm, 7 mm, 12 mm, 17 mm and 22 mm, respectively. The shaker holds one side for excitation, and the fixture holds the other to give a fixed boundary condition. Then the laser Doppler vibrometers (LDV) are placed to measure the velocities at the excitation point and the center of the cavity.

The experiment process is as follows. First, the input sine signal is generated by the function generator (33210A, Keysight), and the generated signal is amplified by the amplifier (type 2718, B&K) based on the signal. The shaker (type 4809, B&K) induces a harmonic excitation to the side beam of the metamaterial system. The time-velocity data is measured by two LDV; the velocity at the shaker is measured by LDV #1 (CLV-2534, Ploytec), and the velocity at the cavity is measured by LDV #2 (Nova, Optomet). After measurement, the measured signals are post-processed by the fast Fourier transformation to extract the amplitude of the excitation frequency component. Finally, the extracted velocity amplitude at the cavity *A*_*c*_ is divided by the amplitude at the shaker *A*_*s*_ to obtain the amplification ratio for each cavity length of 60 mm, 80 mm, 100 mm, 120 mm and 140 mm. In general, impulse signal or frequency swap signal are used in such vibration experiments^31^. However, in the current metamaterial cavity, the leaky waves are involved so steady-state should be guaranteed for each frequency. Thus, instead of the impulse or frequency sweep signals, sine signal with a single frequency is used, and the experiment is repeated for various frequencies. The experiment is conducted from 100 to 3500 Hz for every 100 Hz. Around the resonance frequency, the experiment is carried out with a finer frequency interval to precisely measure the resonance behavior.

**Figure 7:**
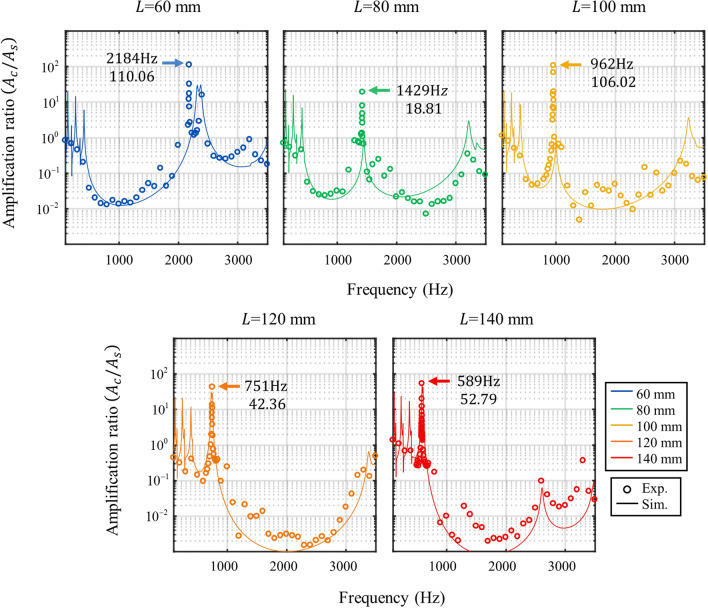
Experimentally (colored circle) and numerically (colored line) obtained amplification ratio of the metamaterial with the optimized side beam for cavity length *L* = 60 mm, 80 mm, 100 mm, 120 mm and 140 mm.

Figure 7 plots the amplification ratio, calculated by dividing the velocity amplitude at the cavity by the amplitude at the shaker. The solid lines indicate simulation results, and the hollow circle makers indicate experimental results. The experimental results and simulation results show good agreement with each other. Focusing on the cavity’s resonance frequencies, the experimentally measured resonance frequencies are 2184 Hz, 1429 Hz, 962 Hz, 751 Hz and 589 Hz for *L* = 60 mm, 80 mm, 100 mm, 120 mm and 140 mm, respectively. These are almost identical to the numerically predicted frequencies 2317 Hz, 1450 Hz, 1002 Hz, 747 Hz and 592 Hz for *L* = 60 mm, 80 mm, 100 mm, 120 mm and 140 mm, respectively. In addition, the amplification ratio of the cavity also shows that the vibration energy is highly localized inside the cavity so that it reaches 110.06, 18.81, 106.02, 42.36 and 52.79 for the cases of the cavity length *L* = 60 mm, 80 mm, 100 mm, 120 mm and 140 mm, respectively.

**Figure 8:**
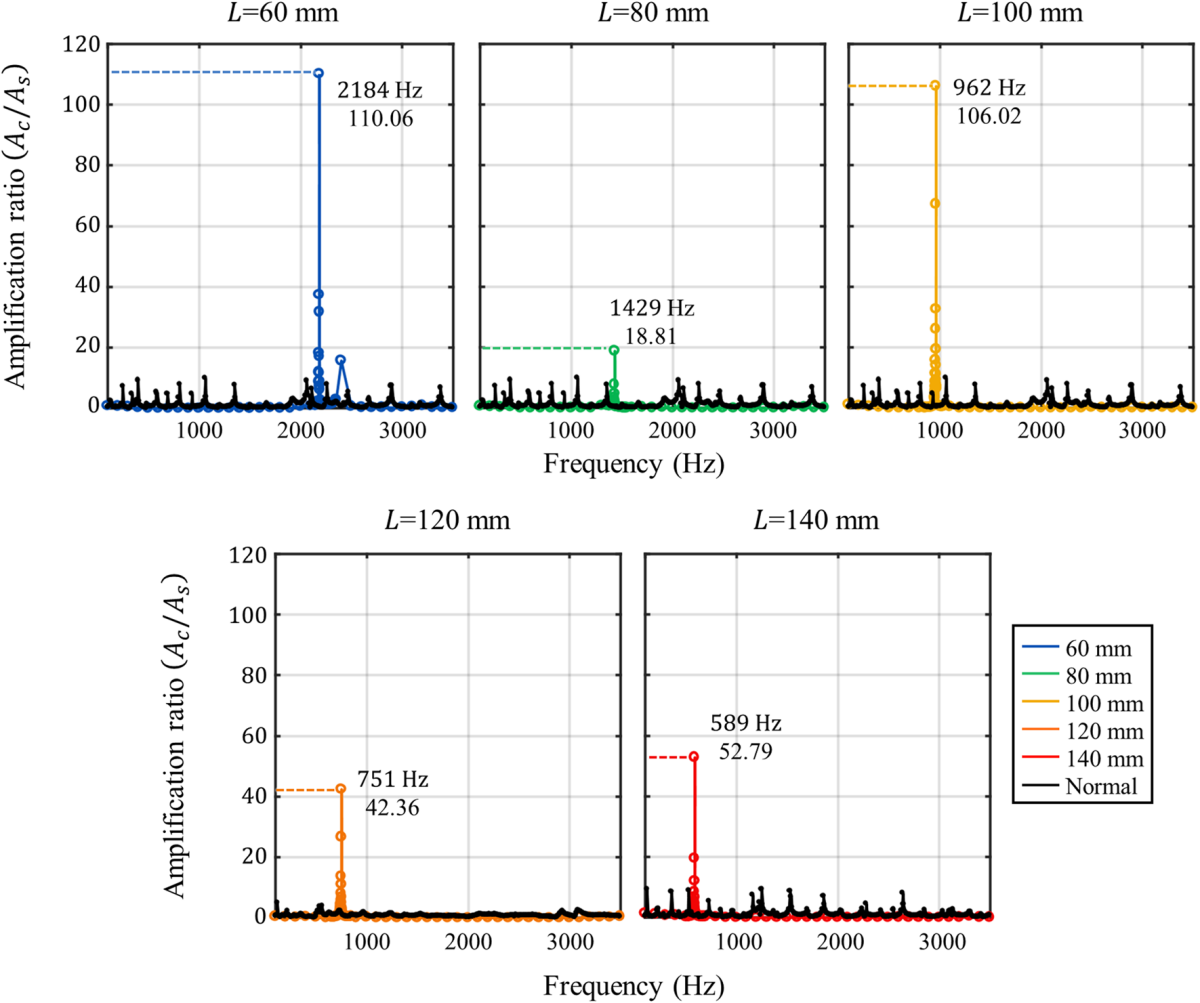
Experimentally obtained amplification ratio of the metamaterial (colored line) with the optimized side beam and the ratio of the normal beam (black line).

To compare the amplification ratio of the metamaterial cavities, we carried out additional experiments with normal aluminum beams. It should be noted that the performance of the cavity is hard to be directly compared with the normal aluminum beams since the two cases have different resonance frequencies. Thus, previous research has focused on the amplitude difference with and without the metamaterial cavities at the same bandgap frequency. Here, we used the same approach; we newly carried out experiments with normal aluminum beams with the same thickness, width and length as the metamaterial cavities at the same frequency range. In Fig. 8, the experimental data of the normal beam is plotted as a black line and the metamaterial cavity is plotted as a colored line. Although there are various resonance peaks in the normal beams, vibration energy is not highly localized; mostly, the amplification ratio does not exceed 10. Thus, all the resonance peaks are much smaller than the metamaterial cavity’s peak where the vibration energy is highly localized inside the cavity.

From those experimental results in this section, it can be seen that the cavity modes with various frequencies within the proposed metamaterial system are successfully validated. Again, we would like to emphasize that the same metamaterial unit is used for all metamaterial cavities. Based on this metamaterial system, one may easily obtain a cavity mode at the desired frequency with the optimized amplitude by simply adjusting the length of the cavity and side beam without re-designing.

## Conclusion

In this paper, we proposed the metamaterial system that implements a cavity mode at a low frequency to localize vibration energy inside the cavity at various frequencies. We designed the metamaterial unit cell consisting of the hollow cylindrical shaped mass and bow-tie shaped spring to realize a broad and low-frequency bandgap based on the analysis of flexural wave propagation using the extended mass-spring system. With this unit cell design, the broad and low-frequency from 522 to 3117 Hz bandgap is obtained. Consequently, the cavity modes of the various frequencies, 2184 Hz, 1429 Hz, 962 Hz, 751 Hz, and 589 Hz for *L* = 60 mm, 80 mm, 100 mm, 120 mm, and 140 mm, are experimentally obtained by simply adjusting the cavity length without changing the unit cell design at a low-frequency regime. In addition, we found the way to optimize the cavity’s performance by using the evanescent coupling with the side beams. Accordingly, the side beam is added to the metamaterial to enhance the amplification of the cavity mode. The resonance of the side beam can be transferred to the cavity across the bandgap as an evanescent mode. Based on this, the amplification performance of the cavity mode can be improved with the side beam of the proper length. As a result, very large displacement amplifications, 110.06, 18.81, 106.02, 42.36, and 52.79, are experimentally observed for cavity length *L* = 60 mm, 80 mm, 100 mm, 120 mm and 140 mm with the side beam of length 3 mm, 7 mm, 12 mm, 17 mm, and 22 mm.

Our metamaterial system provides a method for easily achieving a cavity mode at the desired frequency based on the broad bandgap. Due to the broad bandgap, the cavity mode frequency can be easily shifted by adjusting the cavity length. In the previous works, which mainly focused on a single cavity mode frequency, the cavity mode should have a unit cell design to have the bandgap covering itself. However, in this work, the broad bandgap, which allows the cavity mode to have various frequencies without concern about the bandwidth, is obtained through the hollow cylindrical mass and bow-tie shape spring. Furthermore, the effect of the side beam on the metamaterial cavity mode is investigated. It is revealed that the amplified vibration by the resonance of the side beam can be transferred to the cavity, and it can be utilized for enhancing the performance of the cavity mode for vibration localizing. Consequently, based on these two findings, one may obtain a cavity mode at the desired frequency by adjusting cavity length and may improve the amplification by optimizing the side beam length. This framework could be a guideline for utilizing the cavity mode for the vibration application.

## Data Availability

The datasets used and/or analyzed during the current study available from the corresponding author on reasonable request. For the datasets, contact the corresponding author, joohwan.oh@unist.ac.kr.
